# Branched-Chain Amino Acid Negatively Regulates KLF15 Expression via PI3K-AKT Pathway

**DOI:** 10.3389/fphys.2017.00853

**Published:** 2017-10-25

**Authors:** Yunxia Liu, Weibing Dong, Jing Shao, Yibin Wang, Meiyi Zhou, Haipeng Sun

**Affiliations:** Key Laboratory of Cell Differentiation and Apoptosis of Chinese Ministry of Education, Department of Pathophysiology, Shanghai Jiao Tong University School of Medicine, Shanghai, China

**Keywords:** BCAA, Klf15, PI3K, Akt, regulation

## Abstract

Recent studies have linked branched-chain amino acid (BCAA) with numerous metabolic diseases. However, the molecular basis of BCAA's roles in metabolic regulation remains to be established. KLF15 (Krüppel-like factor 15) is a transcription factor and master regulator of glycemic, lipid, and amino acids metabolism. In the present study, we found high concentrations of BCAA suppressed KLF15 expression while BCAA starvation induced KLF15 expression, suggesting KLF15 expression is negatively controlled by BCAA.Interestingly, BCAA starvation induced PI3K-AKT signaling. KLF15 induction by BCAA starvation was blocked by PI3K and AKT inhibitors, indicating the activation of PI3K-AKT signaling pathway mediated the KLF15 induction. BCAA regulated KLF15 expression at transcriptional level but not post-transcriptional level. However, BCAA starvation failed to increase the KLF15-promoter-driven luciferase expression, suggesting KLF15 promoter activity was not directly controlled by BCAA. Finally, fasting reduced BCAA abundance in mice and KLF15 expression was dramatically induced in muscle and white adipose tissue, but not in liver. Together, these data demonstrated BCAA negatively regulated KLF15 expression, suggesting a novel molecular mechanism underlying BCAA's multiple functions in metabolic regulation.

## Introduction

Branched chain amino acid (BCAA), including leucine, isoleucine, and valine, are essential amino acids. In addition to building proteins, numerous metabolic functions of BCAA have been investigated (Nair and Short, 2005). BCAA modulates insulin and glucagon secretion in pancreas (Meijer and Dubbelhuis, 2004). Increasing BCAA intake was often associated with positive effects on body weight and glucose homeostasis, attenuating obesity and type 2 diabetes mellitus T2DM (Lynch and Adams, 2014). BCAA regulates protein synthesis and degradation in various tissues. In addition, BCAA influences brain function by participating in synthesis and transport of glutamate, an important excitatory neurotransmitter, and by modifying large, neutral amino acid transport at the blood–brain barrier (Joshi et al., 2006).

Branched chain amino acid (BCAA) has recently been linked with numerous diseases. Elevated circulating levels of BCAA have been associated with an increased risk of T2DM and insulin resistance in humans (Newgard et al., 2009; Huang et al., 2011; Melnik, 2012; Lu et al., 2013; Lynch and Adams, 2014). Defect of BCAA catabolism promotes heart failure progression and associated with cardiovascular diseases (Shah et al., 2010; Sun et al., 2016). Impaired BCAA transportation and metabolism is associated with autism (Novarino et al., 2012; Târlungeanu et al., 2016). Elevated plasma BCAA levels have been found in early pancreatic ductal adenocarcinoma patients, supporting formation of non–small cell lung carcinoma and growth of pancreatic ductal adenocarcinoma (Mayers et al., 2014, 2016; Dey et al., 2017). The novel roles of BCAA in metabolic, cardiovascular, neurological diseases, and cancer are being investigated.

Little is known about the molecular basis of BCAA's function in cells. BCAA, especially leucine, is one key regulator of target of rapamycin complex 1 (TORC1) signaling, which is the central component of a complex signaling network of cell growth and metabolism (Saxton and Sabatini, 2017). Recently, SESN2 has been reported to be a cytoplasmic leucine sensor to activate TORC1 (Saxton et al., 2016; Wolfson et al., 2016). It has been implicated that BCAA enhanced TORC1 activation and contributed to insulin resistance (Newgard et al., 2009). In Maple Syrup Urine Disease and heart failure, branched-chain alpha keto acid (BCKA), the intermediate metabolite of BCAA catabolism, suppressed mitochondrial respiration and induced oxidative stress in cells (Funchal et al., 2006; Ribeiro et al., 2007; Sun et al., 2016). Other than these observations, the molecular basis of BCAA's roles in metabolic regulation remains to be fully explored.

KLF15 (Krüppel-like factor 15) is a transcription factor and a master regulator of metabolism. KLF15-deficient mice have impaired ability to catabolize muscle BCAAs as fuel for gluconeogenic flux during fasting (Gray et al., 2007). In addition, during sustained aerobic exercise, KLF15-deficient mice fail to augment muscle lipid utilization and consequently have impaired endurance exercise capacity (Haldar et al., 2012; Prosdocimo et al., 2014). During adipocyte differentiation, KLF15 is dramatically induced and indispensable for the differentiation (Mori et al., 2005). Under fasting condition, KLF15 is induced and inhibits the expression of SREBP-1C in liver, suppressing lipogenesis (Takeuchi et al., 2016). KLF15 regulates the insulin-sensitive glucose transporter GLUT4 expression (Gray et al., 2002). KLF15 is one key regulator of circadian homeostasis and controls the homeostasis of amino acids during circadian cycle (Jeyaraj et al., 2012; Zhang et al., 2015). In addition, KLF15 controlled the circadian homeostasis of bile acid synthesis. KLF15 deficient mice produced much less bile acids that slowed down the absorption of lipid and other nutrients (Han et al., 2015). Thus, KLF15 is a master regulator of circadian and metabolic regulation of bile acid, glycemic, lipid, and amino acids.

In an effort to understand molecular basis of BCAA's function in cells, we examined the gene expression profiles in mouse embryonic fibroblasts (MEF) in response to BCAA starvation and replenishment. Unexpectedly, KLF15 was among the dramatically regulated genes. In the present study, we investigated the KLF15 regulation by BCAA. The results demonstrated BCAA controls KLF15 expression through PI3K-AKT signaling pathway.

## Materials and methods

### Animals

Wildtype C57BL/6 mice were purchased from SLAC Laboratory Animals Company Limited, Shanghai, China. All animals were housed at with a 12-h light, 12-h dark cycle with free access to water and standard chow. This study was carried out in accordance with the guidelines of the Committee for Humane Treatment of Animals at Shanghai Jiao Tong University School of Medicine. The protocol was approved by the Committee for Humane Treatment of Animals at Shanghai Jiao Tong University School of Medicine.

### Cell culture

HepG2, NIH 3T3, and MEF cells were cultured in Dulbecco's modified Eagle's medium (Hyclone, Beijing) supplemented with 10% fetal bovine serum (FBS, Gibco BRL, Gaithersburg, MD), penicillin (100 IU/mL) and streptomycin (100 μg/mL) in a humidified 5% CO2-95% air incubator at 37°C. Custom BCAA-free DMEM was provided by Invitrogen. BCAA and BCKA chemicals were purchased from Sigma.

### Plasmids, transfection, and luciferase assays

The 3′UTR of mouse *KLF15* was identified using the UCSC Genome Browser (http://genome.ucsc.edu/) and cloned into the psiCHECK_2 plasmid (Promega) with XhoI and NotI sites, respectively. The 3'UTR fragment (921 bp) was located in the 3′ flanking region of the synthetic Renilla luciferase gene. Thus, Renilla luciferase expression can be regulated by the downstream 3′UTR activity. The promoter region of *Klf15* (−5 kb before the translation start site in the second exon) was cloned into PGL3 reporter vector (Promega) (Jeyaraj et al., 2012). Plasmids were transfected using lipofectimine2000 (Invitrogen) according to the manufacturer's protocol. The transfected cells were rinsed once with cold PBS and lysed with passive lysis buffer (Promega) after transfection for 24 h. Luciferase activity was measured using the GloMax-Multi Detection System (Promega) and the Dual-Luciferase Reporter Assay System (Promega). Renilla luciferase expression was normalized to the expression of firefly luciferase. Data are represented as means ± SE from three biological replicates representing three independent experiments.

### RNA extraction, reverse transcription and real-time PCR analysis

Total RNA was extracted using the Trizol (Invitrogen) according to the manufacturer's instructions. Total RNA (2 μg) was reverse transcribed using random primers and MMLV (Promega). Each cDNA sample was analyzed in triplicate with the Applied Biosystems Prism7900HT Real-Time PCR System using Absolute SYBR Green (ABI) with the specific primers. The relative amount of specific mRNA was normalized by 18sRNA. Mouse KLF15 primer sequences are: forward, 5′-TCTCGTCACCGAAATGCTCA-3′ and reverse, 5′-GAGTCAGGGCTGGCACAAGA-3′.

### Western blot analysis

Proteins from tissue or cells were harvested in buffer (50 mM HEPES [pH7.4], 150 mM NaCl, 1% NP-40, 1 mM EDTA, 1 mM EGTA, 1 mM glycerophosphate, 2.5 mM sodium pyrophosphate 1 mM Na3VO4, 20 mM NaF, 1 mM phenylmethylsulfonyl fluoride, 1 μg/mL of aprotinin, leupeptin, and pepstatin). Samples were separated on 4–12% Bis-Tris gels (Invitrogen), and transferred onto a nitrocellulose blot (Amersham). The blot was probed with the indicated primary antibodies. Protein signals were detected using conjugated secondary antibodies and enhanced chemiluminescence (ECL) western blotting detection regents (Pierce). The BCKDK antibody (AV52131) was purchased from sigma. Phospho-Akt (#4051 for pAKT Ser473 and #2965 for pAKT Thr308) and total AKT (#9272 for AKT1,2,3) antibodies were purchased from Cell Signaling Technology. β-actin antibody was purchased from Sigma Chemical Co.

### Determination of BCAA concentration

Fifty microliter of plasma was precleared of protein by addition of an equal volume of methanol, followed by two rounds of centrifugation to precipitate protein. The supernatant (90% recovery) was lyophilized and resuspended in 45 μl dH_2_O. Standards (L-valine, L-leucine, L-isoleucine, L-allo-isoleucine) were prepared from powder. 10 mM stocks were made in dH_2_O and serial pooled dilutions prepared. A 250 μM stock of D3-Leucine was also prepared in dH_2_O. 2 μl of standard or 2 μl of sample was diluted in 50 μl of butanolic HCL in 1.5 ml tube, heated to 60°C for 20 min and then speed vacuumed to dryness. The samples were resuspended in 200 ul of 50:50 dH_2_O:acetonitrile containing 0.1% formic acid. The samples were analyzed by LC-MS/MS.

### Statistical analysis

Unless otherwise specified, statistical analyses were performed with Student's *t*-test (two groups) or one-way ANOVA (>2 groups) where appropriate. Data are calculated as the mean ± SE (standard error) unless otherwise indicated. A *p*-value of less than 0.05 was considered statistically significant.

## Results

### KLF15 expression is negatively controlled by BCAAs

**Figure 1:**
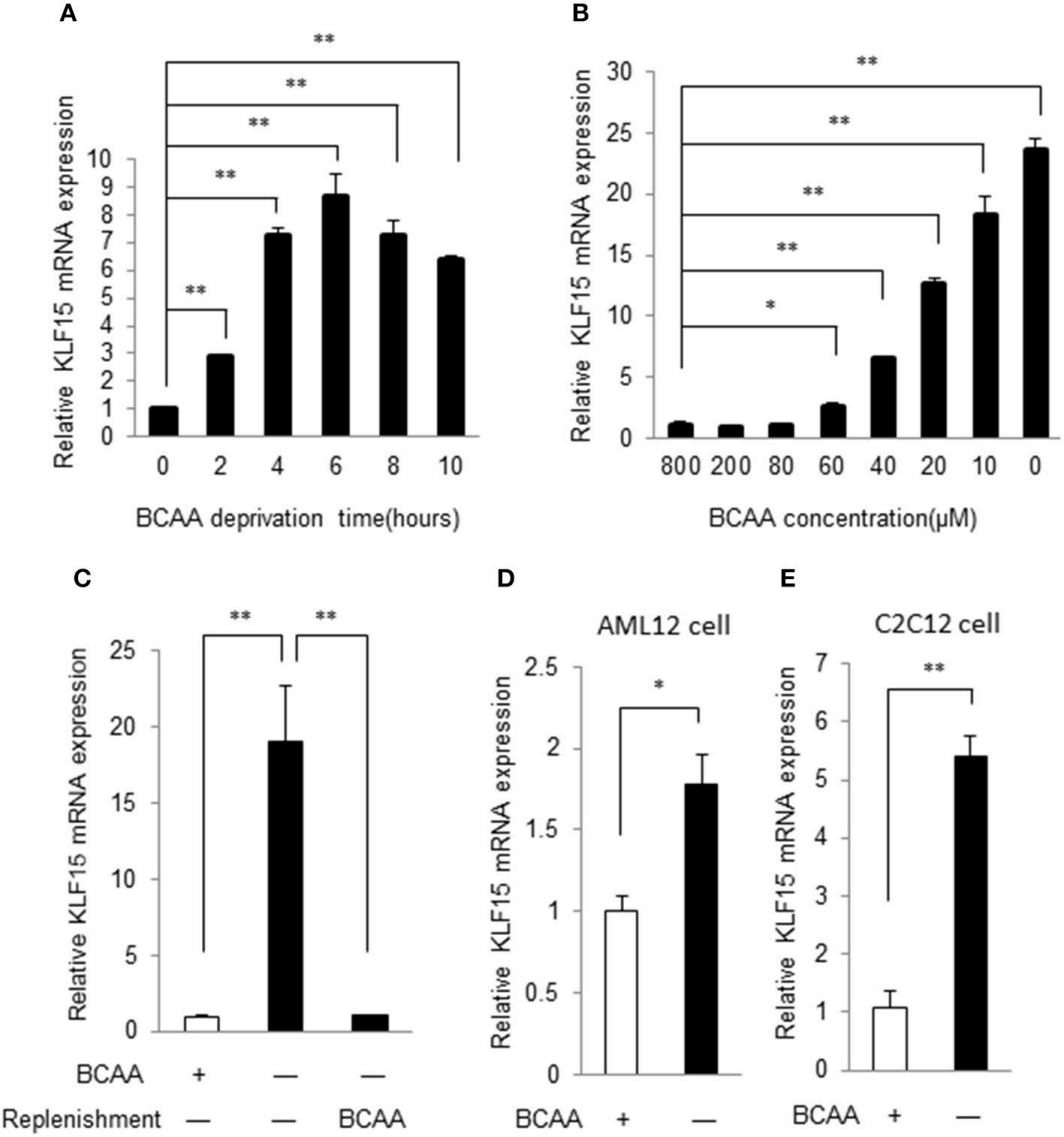
BCAA negatively controls KLF15 expression. Quantitative PCR results of KLF15 in MEFs. **(A)** MEFs were cultured in BCAA-free DMEM for indicated time before harvesting. **(B)** MEFs were cultured with different concentrations of BCAAs for 6 h before harvesting. **(C)** MEFs were cultured without BCAA (−) for 4 h followed with or without (−) BCAA replenishment for extra 2 h before harvesting. **(D,E)**, AML12 liver cell line **(D)** and C2C12 muscle cell line **(E)** were cultured with (+) or without (−) BCAA for 4 h before harvesting. Data are means ± SE for three individual samples. ^*^p < 0.05, ^**^p < 0.01.

To verify the KLF15 regulation by BCAAs identified in gene expression profiles of MEF (data not shown), we examined the time course of KLF15 expression following BCAA deprivation. In commercial Dulbecco's Modified Eagle's Medium (DMEM), the BCAAs concentration is 800 μM. A customized DMEM containing all components except for BCAAs was used to deprive BCAA. In MEFs, KLF15 mRNA demonstrated a clear increase at 2 h after BCAA deprivation, and remained elevated for at least 10 h (Figure 1A). Further study showed that BCAA suppressed KLF15 expression in a dose-dependent way (Figure 1B). To further verify the suppression of BCAA on KLF15 expression, we starved the cells with BCAA-free DMEM and then replenished with BCAA. BCAA replenishment clearly inhibited the BCAA-deprivation-induced KLF15 expression (Figure 1C). KLF15 induction by BCAA starvation was also observed in other cell types, such as liver (AML12) and muscle (C2C12) cell lines (Figures 1D,E).

### BCAA but not their catabolites regulate KLF15

**Figure 2:**
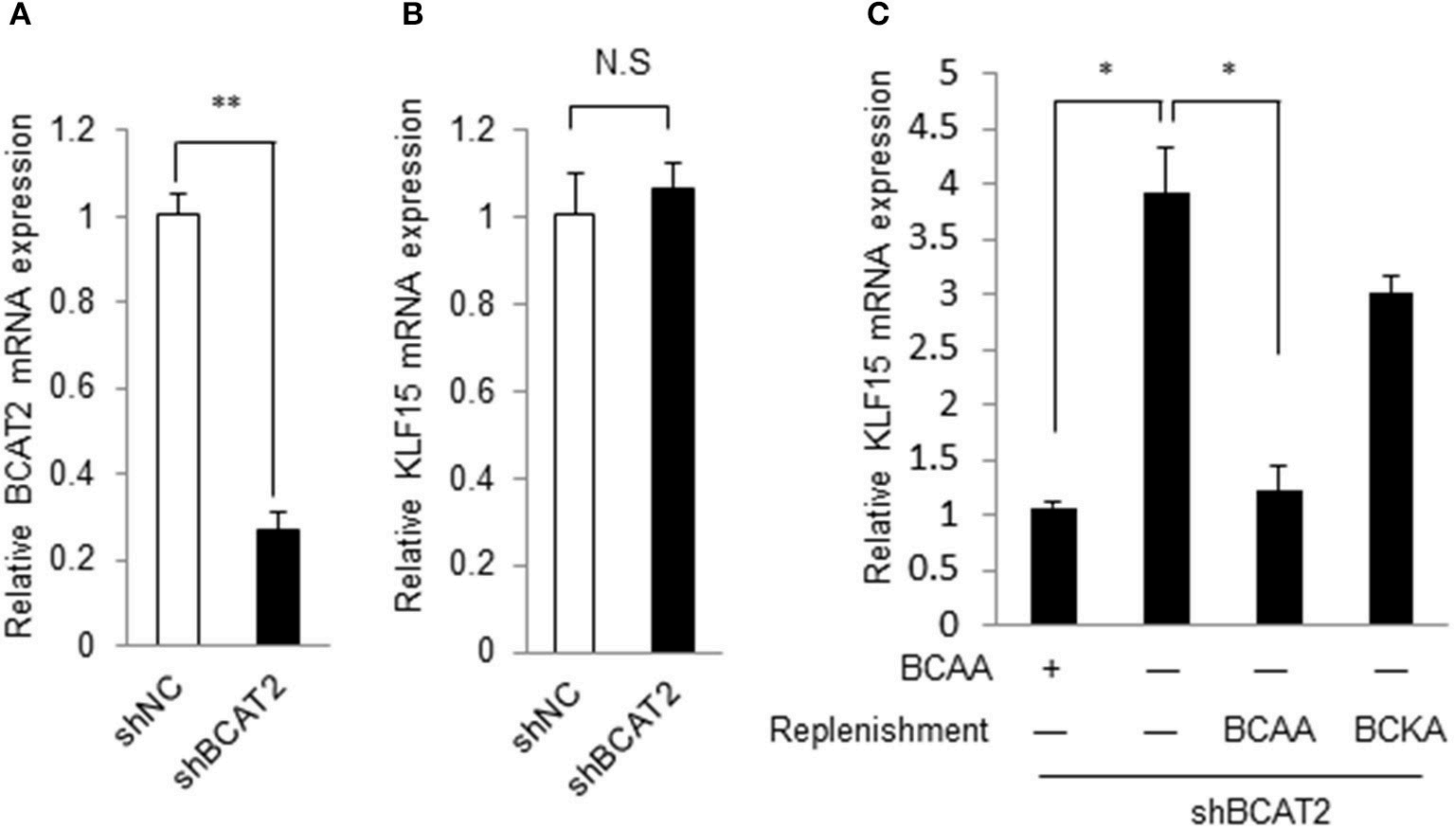
BCAA but not their catabolites regulate KLF15 expression. **(A,B)** Quantitative PCR results of BCAT2 **(A)** or KLF15 **(B)** in MEFs with *Bcat2* silencing using shRNA. **(C)** Quantitative PCR results of KLF15 in MEFs with *Bcat2* silencing with or without BCKA (500 μM) or 800 μM BCAAs replenishment for 2 h after 4-h BCAA starvation (−). ^*^p < 0.05, ^**^p < 0.01.

BCAA can be catabolized in cells. BCAA and their catabolites such as BCKA and 3-hydroxyisobutyrate exert various physiological and pathological functions (Lynch and Adams, 2014; Jang et al., 2016). We then explored whether BCAA or their catabolic intermediates affected the KLF15 expression. Branched-chain amino acid transaminase 2 (BCAT2) catalyzes the first reaction of BCAA catabolism. If the BCAT2 expression is abolished, BCAA can't be catalyzed into BCKA and other intermediates. When BCAT2 expression was silenced by siRNA in NIH-3T3 cell (Figure 2A), KLF15 expression was not affected (Figure 2B). Meanwhile, In the BCAT2 knockdown cell, KLF15 expression was induced by BCAA deprivation, which was inhibited by replenishing BCAA (Figure 2C). Thus, BCAA, but not BCKA or downstream catabolites, suppressed the expression of KLF15.

### BCAA starvation activates PI3K-AKT signaling pathway

**Figure 3:**
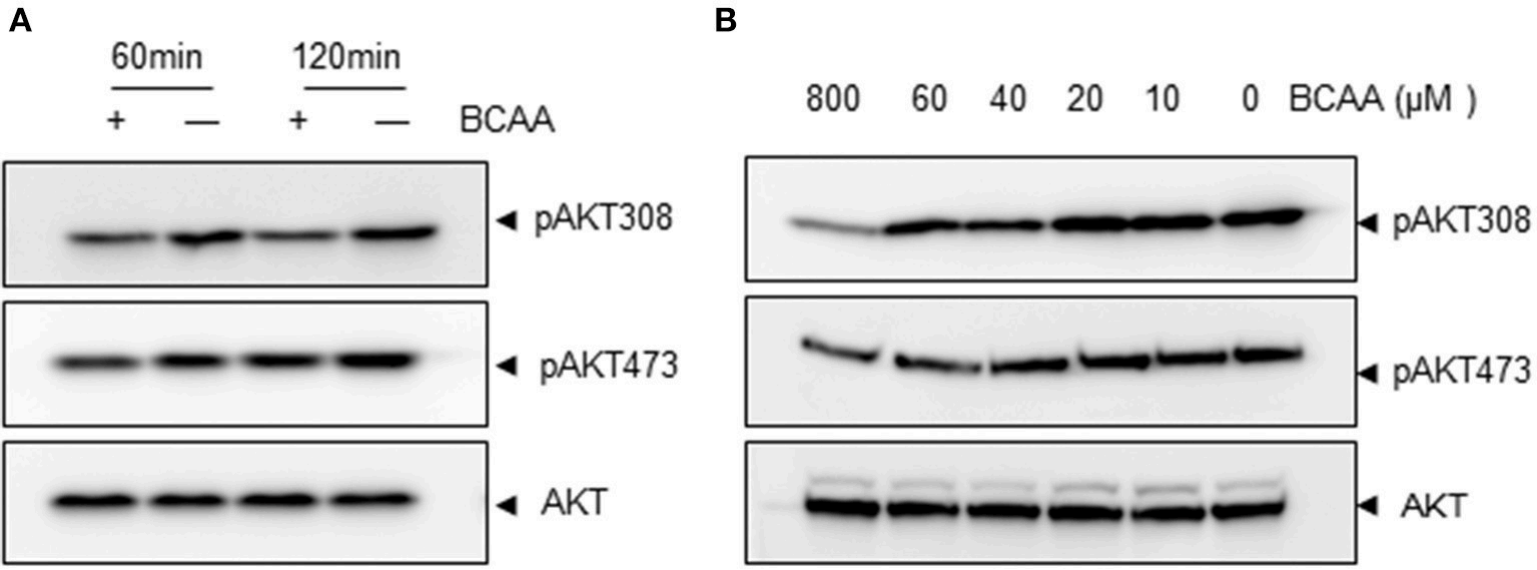
BCAA starvation induces AKT phosphorylation. Measurements of Ser473 or Thr308 phosphorylation of AKT (pAKT) or total AKT (60KD band) using immunoblots. **(A)** MEF cells were treated with BCAA deprivation (−) for indicated time. **(B)** MEF cells were treated with different concentrations of BCAAs for 2 h. Data showed 1 experiment representative of 3.

We then explored the signaling pathway(s) involved in KLF15 regulation by BCAA. A recent study showed that amino acid starvation activated PI3K/AKT signaling (Tato et al., 2011). PI3K is a key signaling molecule to transduce extracellular signals to regulate gene expression in a variety of cell types. AKT is a well-known downstream target of PI3K and its activation can be measured by phosphorylation at signature sites Ser473 or Thr308. To address whether BCAA deprivation activated PI3K pathway, we determined the AKT phosphorylation at Ser473 and Thr308 in response to BCAA starvation. BCAA deprivation caused significant AKT activation at 1 and 2 h (Figure 3A; Figure S1). BCAAs deprivation induced AKT activation in a dose-dependent way (Figure 3B; Figure S2).

### PI3K-AKT signaling mediates BCAA-starvation-induced KLF15 expression

**Figure 4:**
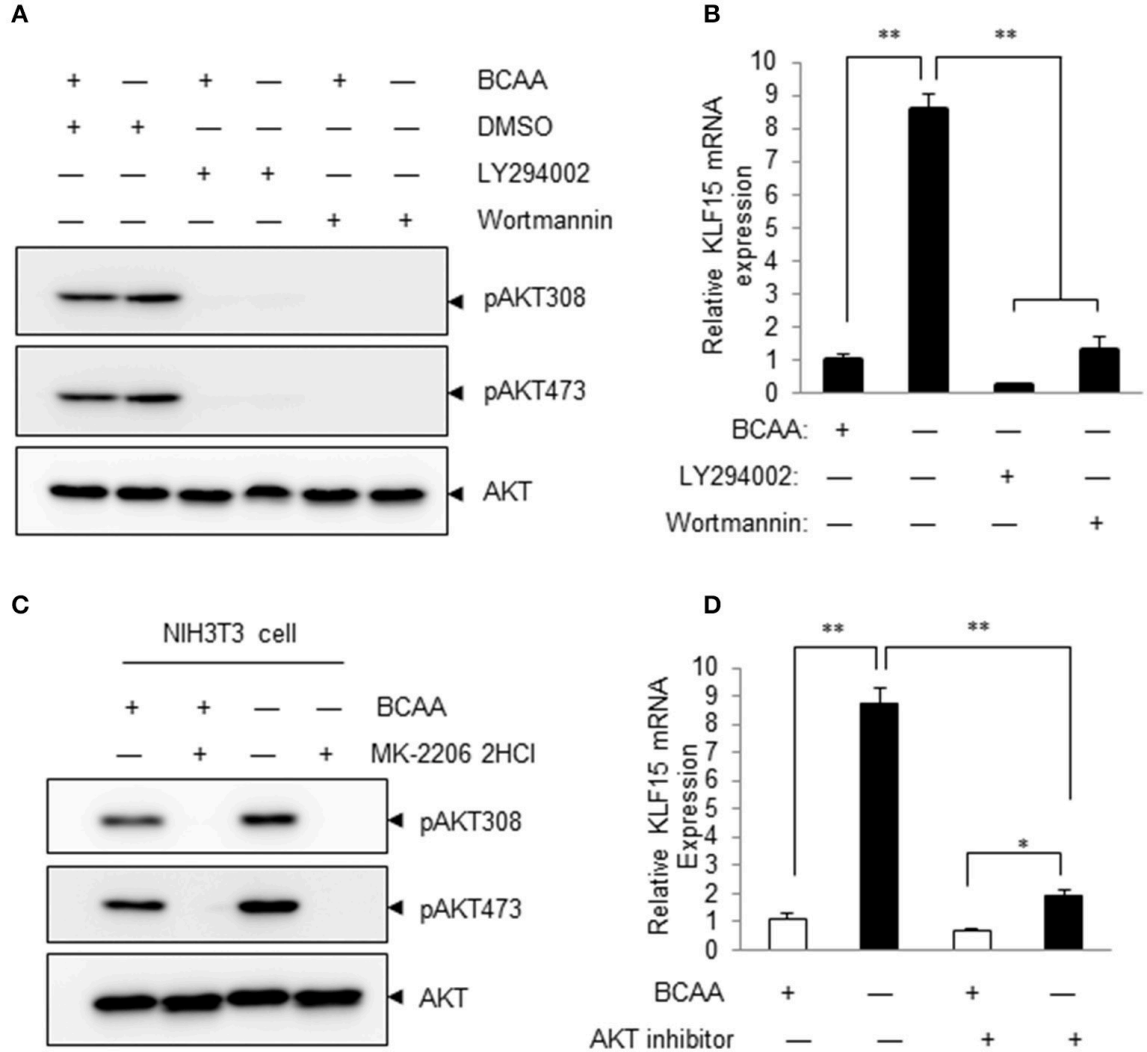
Inhibitors of PI3K and AKT attenuate BCAA-starvation-induced KLF15 expression. **(A)** Measurements of Ser473 or Thr308 phosphorylation of AKT (pAKT) or total AKT (60KD band) using immunoblots. Cells were pretreated 60 min with 10 μM LY294002 or 1 μM Wortmannin before BCAA deprivation (−) for 2 h. Data showed 1 experiment representative of 3. **(B)** Cells were pretreated 60 min with 10 μM LY294002 or 1 μM Wortmannin before BCAA deprivation (−) for 4 h and then harvested for measurement of KLF15 mRNA by quantitative PCR. **(C)** Measurements of Ser473 or Thr308 phosphorylation of AKT (pAKT) or total AKT (60KD band) using immunoblots. Cells were pretreated 60 min with 10 μM AKT inhibitor MK-2206 before BCAA deprivation (−) for 2 h. Data showed 1 experiment representative of 3. **(D)** Cells were pretreated 60 min with 10 μM AKT inhibitor MK-220 before BCAA deprivation (−) for 4 h and then harvested for measurement of KLF15 mRNA by quantitative PCR. Data are means ± SE for three individual samples. ^*^p < 0.05, ^**^p < 0.01.

Pharmacological inhibitors of PI3K were then used to determine whether PI3K signaling pathway participated in BCAA-deprivation-induced KLF15 expression. The basal level and BCAA-starvation-induced Akt phosphorylation at Thr308 and Ser473 were blocked by LY294002 and Wortmannin, demonstrating a clear inhibition of PI3K signaling (Figure 4A; Figure S3). Both LY294002 and Wortmannin abolished BCAA-starvation-induced KLF15 expression (Figure 4B). Similarly, AKT inhibitor MK-2206 inhibited AKT activation (Figure 4C; Figure S4) and the KLF15 mRNA expression induced by BCAA starvation (Figure 4D). Together, these data support a critical role of PI3K-AKT signaling pathway in BCAA-starvation-induced KLF15 expression.

### BCAA regulates KLF15 expression at transcriptional level

**Figure 5:**
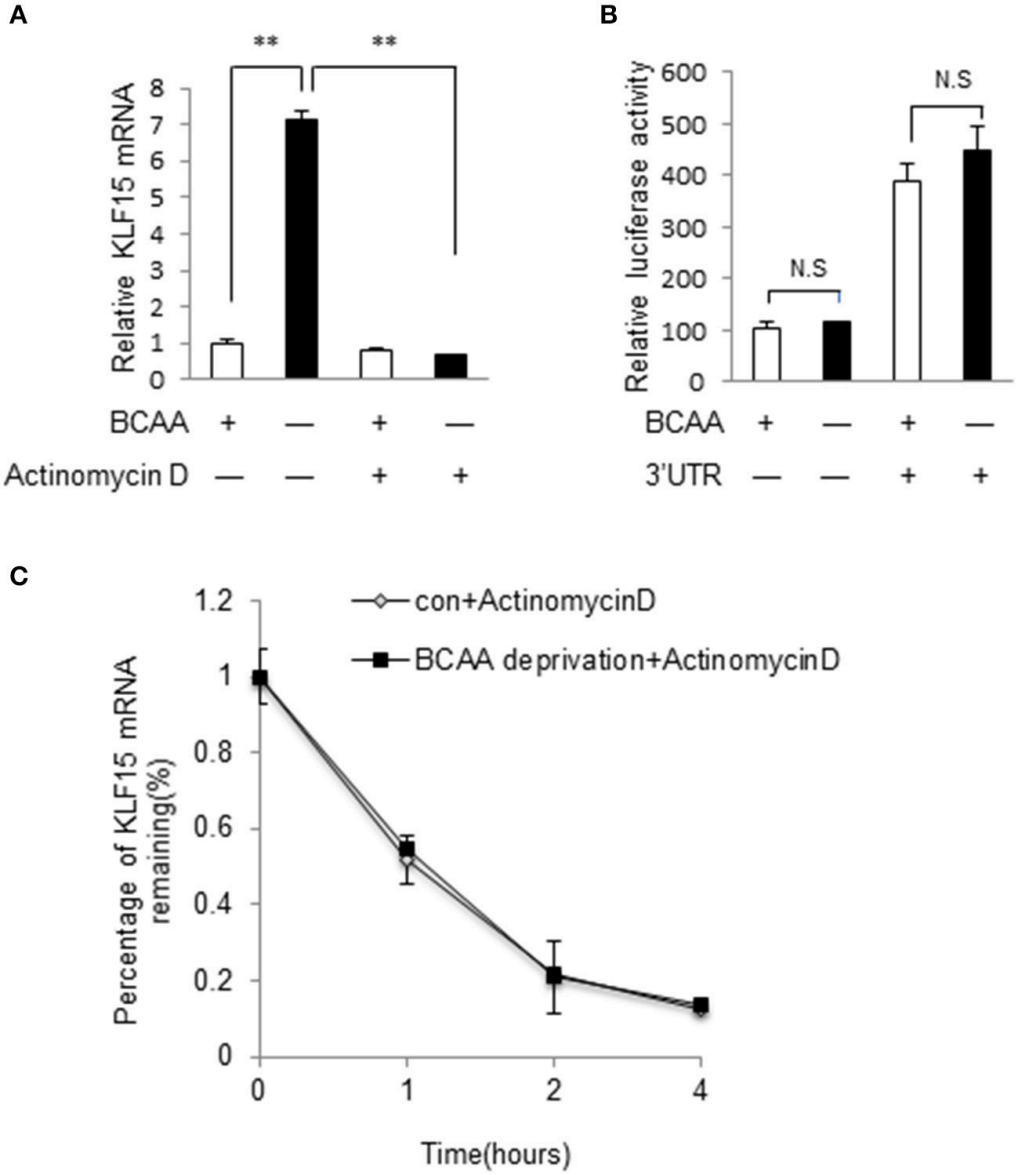
BCAA regulates KLF15 expression at transcriptional level. **(A)** Quantitative PCR was performed to measure the mRNA level of KLF15 in cells pretreated with actinomycin D (5 μg/ml) or DMSO for 30 min before BCAA starvation (−) for 6 h. **(B)** Luciferase assay results from MEF cells transfected with luciferase reporter vectors with (+) or without (−) KLF15 3'UTR with (+) or without BCAA (−). **(C)** Quantitative PCR was performed to measure the relative mRNA level of KLF15 in cells treated with actinomycin D (5 μg/ml) for indicated time in the presence (con) or absence of BCAA (BCAA starvation). Data are means ± SE for three individual samples. ^**^p < 0.01.

We then investigated whether BCAA starvation induced KLF15 expression at transcriptional or post-transcriptional level. Actinomycin D, an inhibitor of RNA synthesis, prevented KLF15 induction by BCAA starvation (Figure 5A), suggesting a transcriptional regulation. To investigate whether BCAAs affected KLF15 expression at post-transcriptional level, we cloned mouse KLF15 3'UTR fragments into the reporter vector p-siCHECK_2, where Renilla luciferase expression is regulated by downstream 3′UTR, and generated Luc-3′UTR reporters. After transfecting this reporter vector into cells, the luciferase activity was measured. The results showed that BCAA starvation did not affect the expression of the luciferase fused with mouse KLF15 3'UTR (Figure 5B). Meanwhile, BCAA starvation failed to affect the half-life of KLF15 mRNA (Figure 5C). These data suggest that BCAA starvation affect the KLF15 expression at transcriptional but not post-transcriptional level.

### BCAA does not directly regulate KLF15 promoter activity

**Figure 6:**
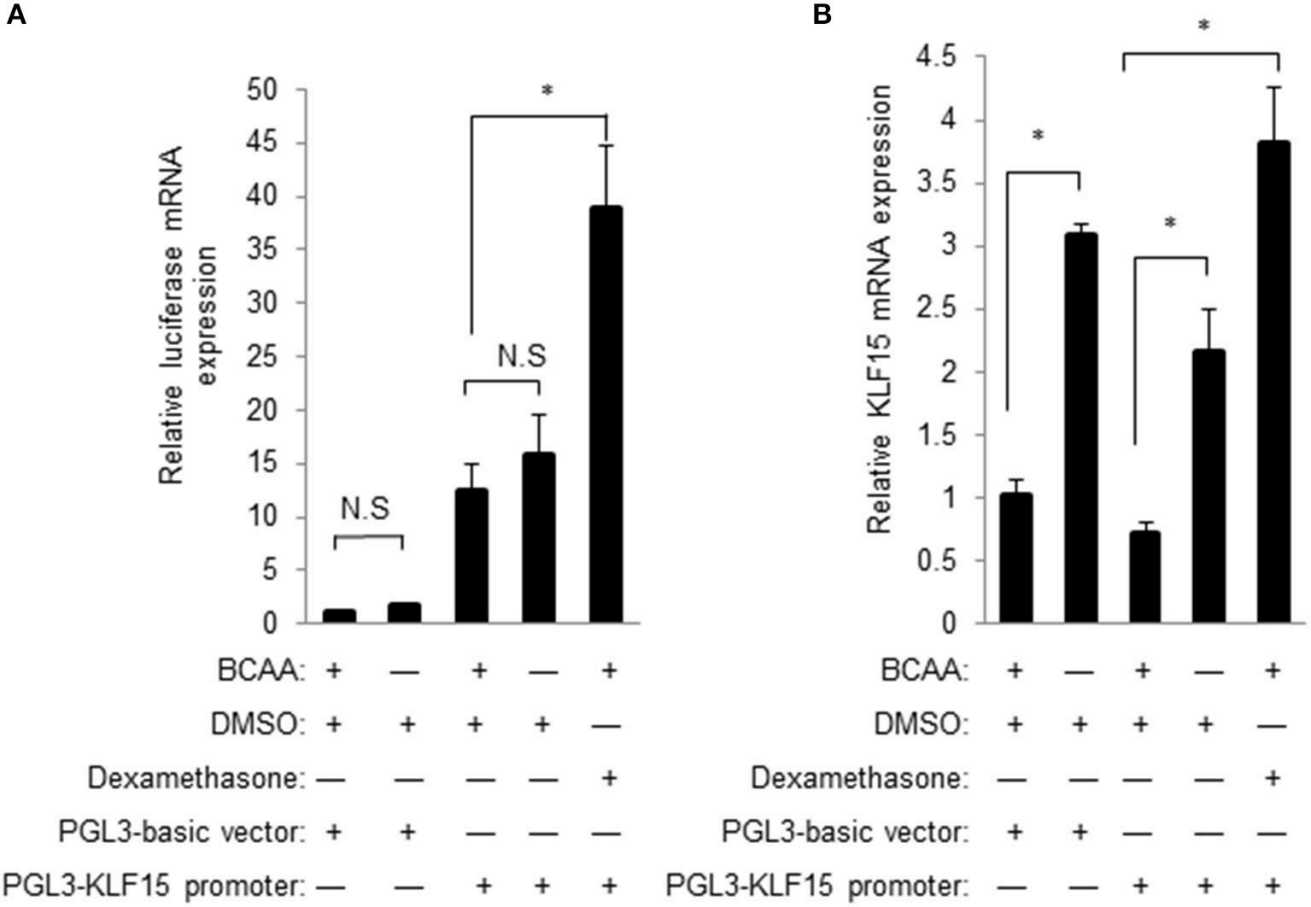
BCAA does not directly affect KLF15 promoter activity. Quantitative PCR was performed to measure the mRNA level of luciferase **(A)** or KLF15 **(B)** in cells transfected with empty PGL3-basic or KLF15-promoter-driving luciferase constructs and treated with BCAA starvation (−) or dexamethasone for 24 h. Data are means ± SE for three individual samples. ^*^p < 0.05.

A common mechanism of transcriptional regulation is promoter activation. A chimeric construct of luciferase gene under the control of KLF15 promoter was introduced into cells by transient transfection (Jeyaraj et al., 2012). KLF15-promoter contains a well-defined GRE for the transactivity by glucocorticoid receptor (Shimizu et al., 2011). Unexpectedly, BCAA starvation failed to increase the KLF15-promoter-driven luciferase activity (data not shown). Meanwhile, the KLF15-promoter-driven luciferase mRNA expression was also not induced by BCAA starvation (Figure 6A) even BCAA starvation induced endogens KLF15 mRNA expression (Figure 6B). On the other hand, activation of KLF15 promoter and mRNA were observed following dexamethasone treatment (Figure 6). Thus, BCAA starvation induced the KLF15 transcription without directly affecting KLF15 promoter activation.

### BCAA abundance is negatively associated with KLF15 expression *in Vivo*

**Figure 7:**
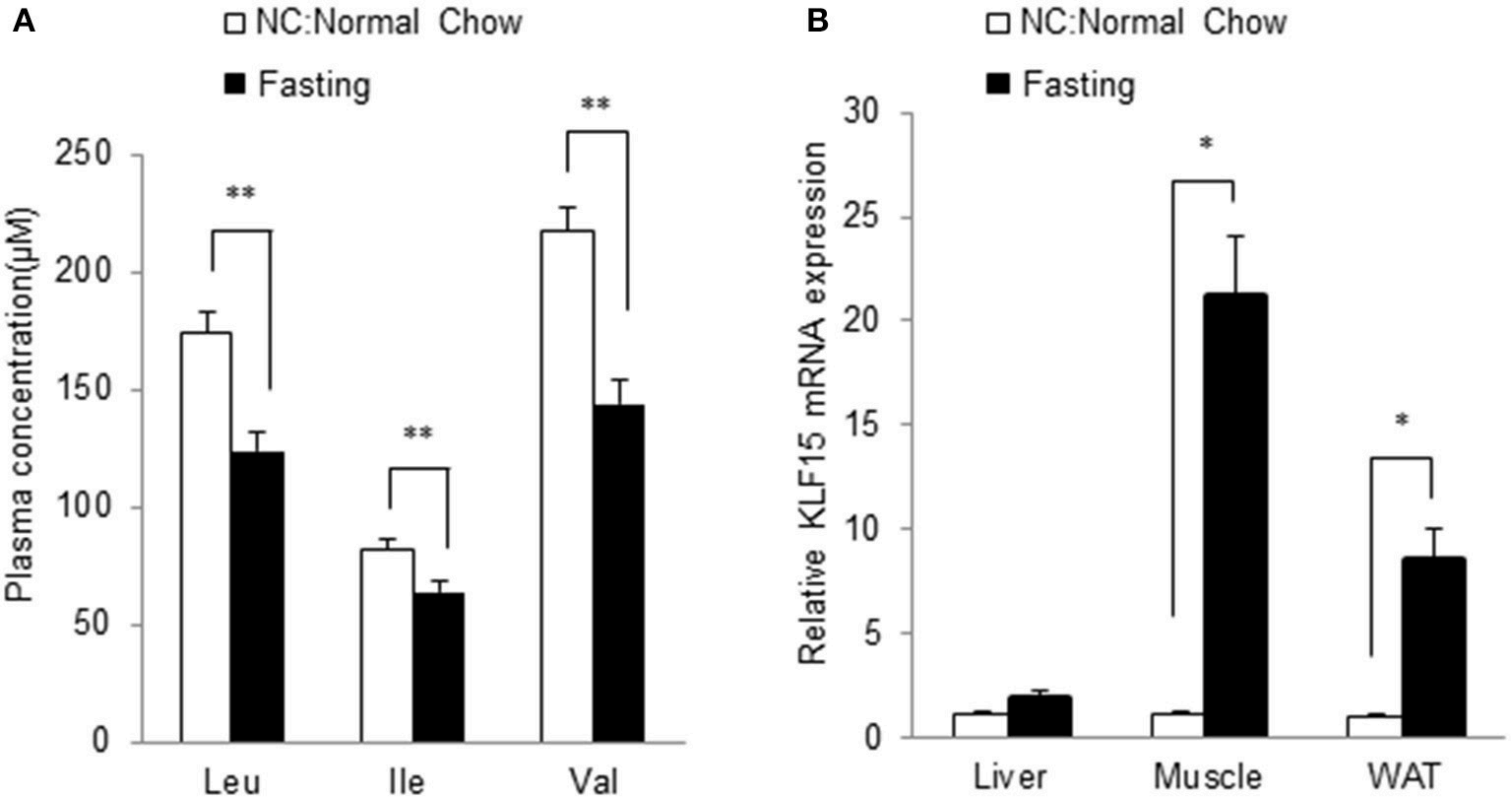
Fasting leads to lower BCAA abundance and higher KLF15 expression *in vivo*. Mice at 10-week of age were fasted for 12 h. Plasma levels of BCAAs were measured (**A**, *n* = 7 in each group) and mRNA level of KLF15 in different tissues were analyzed with quantitative PCR (**B**, *n* = 4 in each group). ^*^p < 0.05, ^**^p < 0.01.

We further investigated the relationship between BCAA and KLF15 expression *in vivo*. Plasma BCAA levels significantly decreased after 12-h fasting (Figure 7A). We measured the KLF15 expression in different tissues in fasted mice. KLF15 expression was significantly induced in muscle and white adipose tissue, but not in liver (Figure 7B). These data suggest that, *in vivo*, BCAA abundance was negatively associated with KLF15 expression.

## Discussion

Our data demonstrated that KLF15 expression is negatively controlled by BCAA. BCAA starvation induced KLF15 expression. The regulation occurs at transcriptional level although KLF15 promoter activity was not directly controlled by BCAA. Interestingly, BCAA starvation induced PI3K-AKT signaling that mediated KLF15 expression. In addition to the cellular studies, we further showed that BCAA abundance is negative associated with KLF15 expression *in vivo*. Considering the critical roles of KLF15 in numerous cellular functions, these data suggested BCAA could play roles in metabolic regulation through controlling KLF15 expression.

It has been well established that BCAA, particularly leucine, positively regulates mTOR activity. The mechanism was revealed only recently, showing leucine directly interacts with its sensor Sestrin2 and activates mTOR via numerous protein-protein interactions on the surface of lysosome (Saxton et al., 2016; Wolfson et al., 2016). In this way, BCAA, particularly leucine, positively regulates TORC1 activity independent of AKT activity. On the other hand, insulin and other growth factors induced mTOR via activating PI3K-AKT pathway (Saxton and Sabatini, 2017). In the present study, BCAA starvation reduced TORC1 activity (data not shown). Therefore, it is interesting to find that BCAA starvation induced PI3K-AKT activity while reducing mTOR activity. Further investigations are warranted to elucidate how BCAA-regulated PI3K-AKT and Sestrin2 pathways crosstalk to determine the mTOR activity.

Our results showed that BCAA regulates KLF15 mRNA expression at transcriptional level, illustrated by the inhibition from actinomycin D and lack of activity by KLF15 3′UTR. However, the KLF15 promoter-driving luciferase expression was not affected by BCAA starvation even though the endogenous KLF15 mRNA is induced. The KLF15 promotor used in the current study has high GC content. It is possible that the structure of KLF15 promoter in the plasmid impaired the response to BCAA starvation. On the other hand, epigenetic changes induced by BAA starvation has been reported (Chaveroux et al., 2010). Amino acid starvation induces the transcription of numerous genes such as CHOP, ATF3, and TRB3, a process associated with modifications of histone acetylation and chromatin structure. The KLF15 promoter activation may require epigenetic changes, which remains to be further explored.

The KLF15 induction in response to BCAA starvation appears to be cell type specific. Among different cell types, liver cell line such as AML12 showed the weakest increase of KLF15 mRNA in response to BCAA starvation. The KLF15 mRNA in AML12 was induced ~1.8-fold by BCAA deprivation, compared to more than 5-fold induction in other cell types (Figure 1). Similarly, in liver, the KLF15 expression showed a trend for increase in response to fasting, whereas the KLF15 induction was more significant in skeletal muscle and white adipose tissue. This cell-type-specific KLF15 induction indicated diverse BCAA sensing mechanisms and responses to BCAA nutrient in different cells and tissues.

In conclusion, our data showed that BCAA negatively regulate KLF15 expression. Dysfunctional BCAA homeostasis has been linked with metabolic, neurological, cancer, and cardiovascular diseases three regulation by BCAA strongly indicates a novel mechanism for BCAA's roles in different physiological processes.

## Ethics statement

All animal procedures were carried out in accordance with the guidelines and protocols approved by the Committee for Humane Treatment of Animals at Shanghai Jiao Tong University School of Medicine.

## Author contributions

HS designed the research; MZ helped to design the overall study; WD, YL, and JS performed the research; MZ, YW, and HS analyzed the data; all authors contributed to the manuscript preparation.

### Conflict of interest statement

The authors declare that the research was conducted in the absence of any commercial or financial relationships that could be construed as a potential conflict of interest.
