# Supplementary figures and images for: Branched-Chain Amino Acid Negatively Regulates KLF15 Expression via PI3K-AKT Pathway
Source: Front Physiol. 2017 Oct 25;8:853. doi: 10.3389/fphys.2017.00853 (PMC5661165; doi:10.3389/fphys.2017.00853)

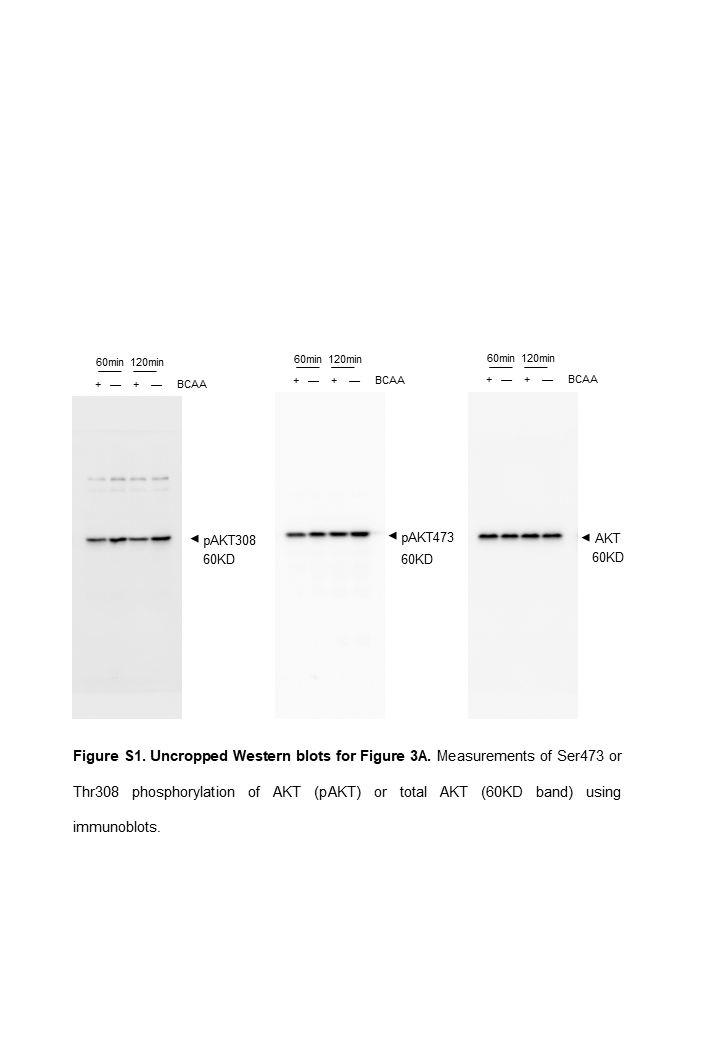

Supplement: Supplementary file 1 [file Image1.tif]

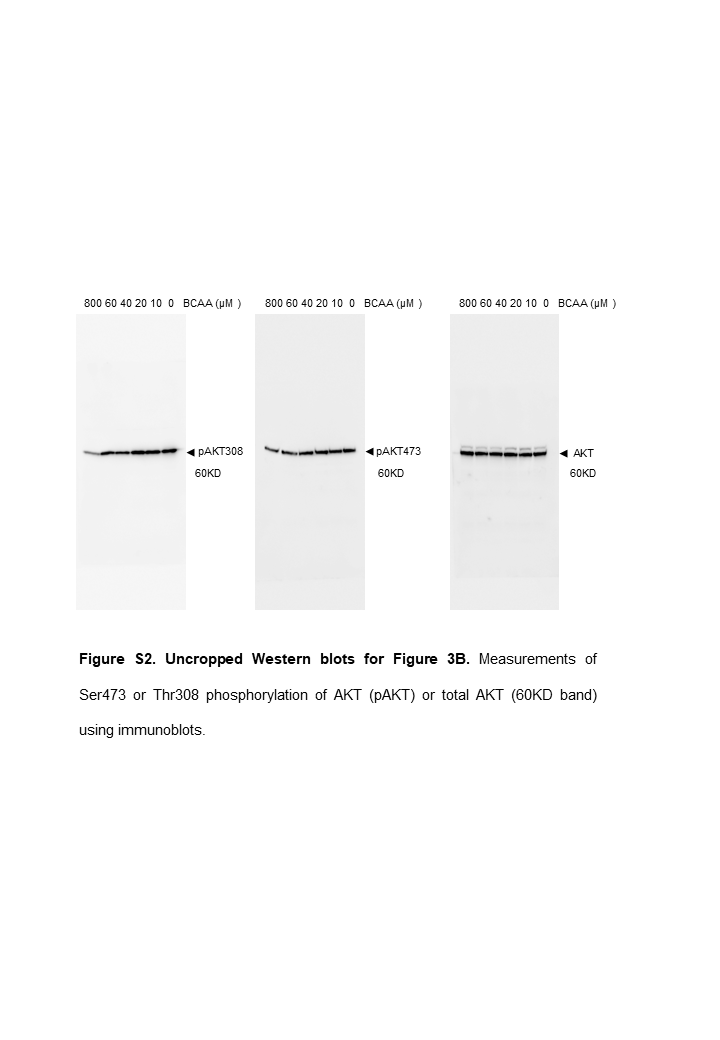

Supplement: Supplementary file 2 [file Image2.tif]

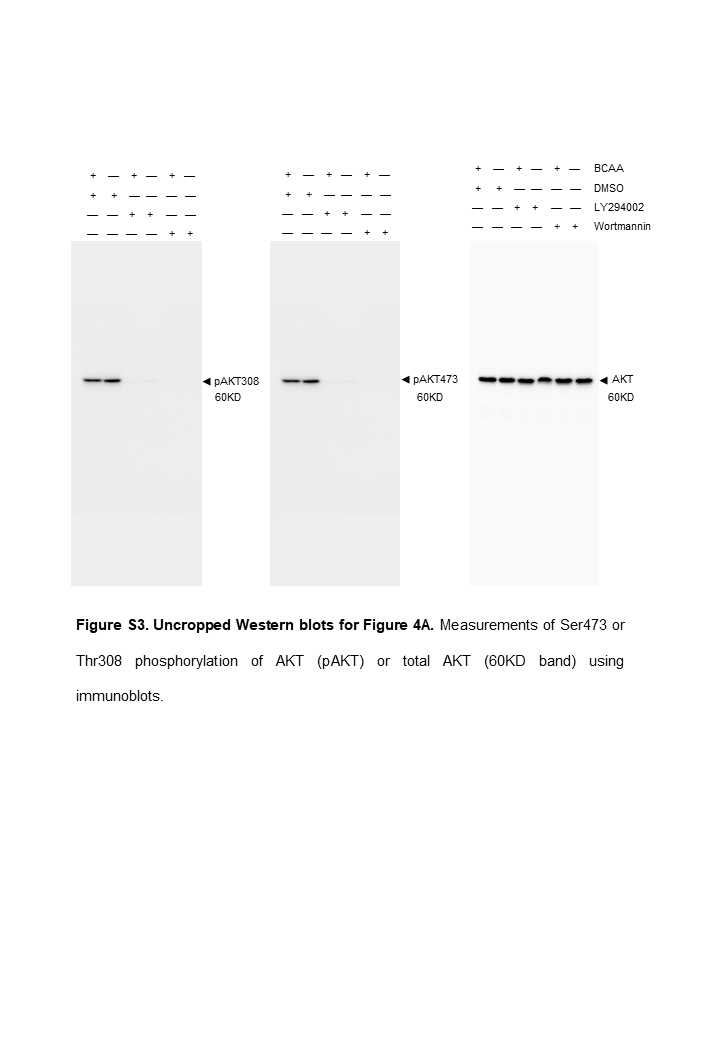

Supplement: Supplementary file 3 [file Image3.tif]

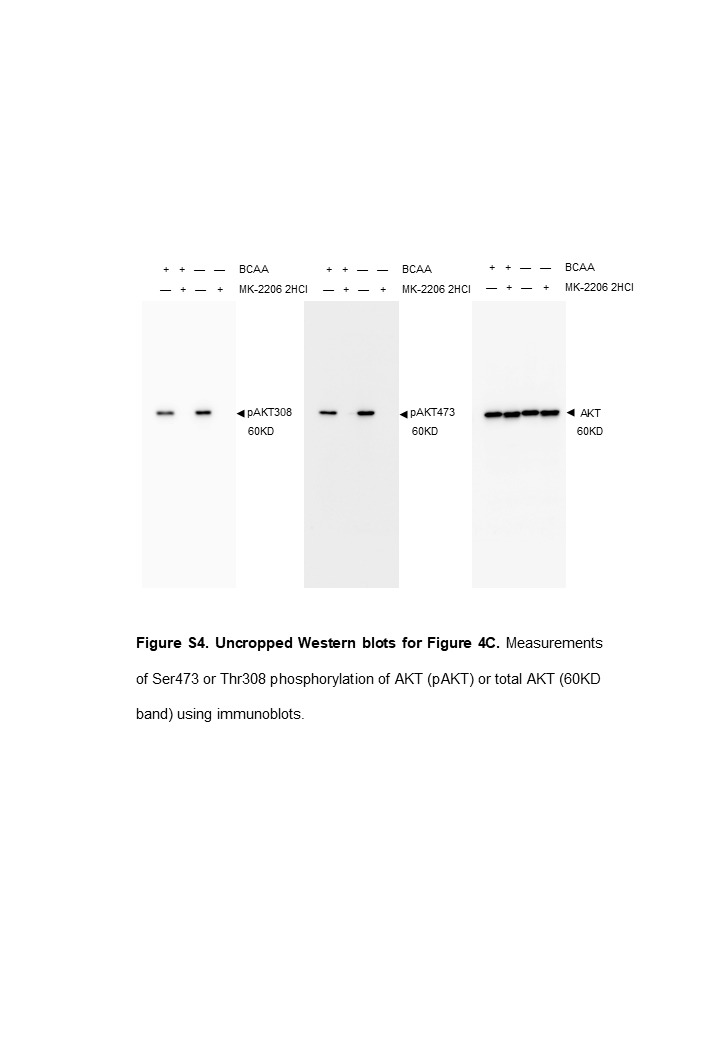

Supplement: Supplementary file 4 [file Image4.tif]
